# Impact of the neutron-depolarization effect on polarized neutron scattering in ferromagnets

**DOI:** 10.1107/S2052252521003249

**Published:** 2021-04-13

**Authors:** Yifan Quan, Jakob Steiner, Victor Ukleev, Joachim Kohlbrecher, Alexei Vorobiev, Patrick Hautle

**Affiliations:** a Laboratory for Neutron and Muon Instrumentation, Paul Scherrer Institute, 5232 Villigen, Switzerland; b Laboratory for Neutron Scattering and Imaging, Paul Scherrer Institute, 5232 Villigen, Switzerland; c Department of Physics and Astronomy, Division of Materials Physics, Uppsala University, Uppsala, Sweden

**Keywords:** materials science, magnetic scattering, magnetic structures, polarized neutron scattering, neutron depolarization, spin-leakage correction, small-angle neutron scattering, ferromagnets

## Abstract

It has been known for decades that a ferromagnetic sample can depolarize a transmitted neutron beam. This effect was used and developed into the neutron-depolarization technique to investigate the magnetic structure of ferromagnetic materials. Since the polarization evolves continuously as the neutrons move through the sample, the initial spin states on scattering will be different at different depths within the sample. This leads to a contamination of the measured spin-dependent neutron-scattering intensities by the other spin-dependent cross sections. The effect has rarely been considered in polarized neutron-scattering experiments even though it has a crucial impact on the observable signal. A model is proposed to describe the depolarization of a neutron beam traversing a ferromagnetic sample, provide the procedure for data correction and give guidelines to choose the optimum sample thickness. It is experimentally verified for a small-angle neutron-scattering geometry with samples of the nanocristalline soft-magnet Vitroperm (Fe_73_Si_16_B_7_Nb_3_Cu_1_). The model is general enough to be adapted to other types of neutron-diffraction experiments and sample geometries.

## Abstract 2

A model is proposed to describe the depolarization of a neutron beam traversing a ferromagnetic sample, provide the procedure for data correction and give guidelines to choose the optimum sample thickness. It is experimentally verified for a small-angle neutron-scattering geometry with samples of the nanocristalline soft-magnet Vitroperm (Fe_73_Si_16_B_7_Nb_3_Cu_1_).

## 1. Introduction   

The effect of neutron depolarization in ferromagnets has been realized and studied since the 1940s and was used to directly investigate magnetic domains [see *e.g.* the following references (Halpern & Holstein, 1941 ▸; Burgy *et al.*, 1950 ▸; Maleev & Ruban, 1972 ▸; Rosman & Rekveldt, 1990 ▸, 1991 ▸; Rekveldt, 1993 ▸; van Wilderen *et al.*, 2002 ▸; Kõszegi *et al.*, 2003 ▸; van Dijk *et al.*, 2004 ▸) and references therein]. The depolarization can be explained by the fact that in ferromagnetic materials the neutron spin will precess about the unaligned magnetic flux density **B**(**r**), which is not parallel to the applied magnetic field **H**_0_ because the magnetization **M**(**r**) has a domain structure and is not aligned to **H**_0_, unless the material is fully saturated. [In the work of Maleev & Ruban (1972 ▸), Maleev interpreted the depolarization effect as a result of the very small-angle neutron scattering (SANS), while in the work of Rosman & Rekveldt (1990 ▸), Rekveldt pointed out that the scattering interpretation is basically equivalent to the elucidation using the Larmor precession approach.] As a consequence, the depolarization studies can yield information about the domain structure of magnetic materials. The method probes a scale from ∼10 nm up to macroscopic dimensions. This overlaps with and is complementary to the SANS technique, which is a particularly powerful technique in investigating magnetic domains, probing a scale roughly from 1 to 100 nm. Besides the static magnetic domain structure, the neutron-depolarization technique can also be applied to study the dynamics of magnetic materials with a time resolution of ∼5 µs, *e.g.* the response of the magnetic domain structure reacting to certain actions such as tension and magnetic field change (van Schaik *et al.*, 1981 ▸; Rekveldt, 1993 ▸).

However, the presented work addresses not the depolarization effect as a method to study magnetic domains but rather its general impact on polarized neutron scattering. In our recent polarized SANS studies of ferromagnetic samples, nanocrystalline Vitroperm (Quan *et al.*, 2020 ▸) and mechanically deformed microcrystalline cobalt (Michels *et al.*, 2019 ▸), we were confronted with the consequences of the depolarization effect in the case where the magnetization of the sample was not fully saturated: the measurements with a given incident neutron-spin state were contaminated by ‘spin leakage’, *i.e.* contributions from the other spin state, owing to the depolarization of the beam. We note that this spin leakage is different from the typically discussed spin leakage that originates from imperfect neutron optics [see *e.g.* Quan *et al.* (2019*a* ▸) (in this reference non-zero *T*_↑↓_ and *T*_↓↑_ transmissions actually denote the total depolarization of the sample but the impact of the sample on the neutron scattering is not considered)]. To the best of our knowledge, the discussion of the depolarization effect has so far been limited to transmission experiments and has not been considered for the case of scattering, except for the spin-echo SANS technique (Rekveldt *et al.*, 2006 ▸).

Here we propose a model to quantify the depolarization and correct the corresponding spin leakage in a SANS experiment. It furthermore helps to optimize the sample thickness leading to an optimum signal-to-noise ratio. The model is general enough to be applied to any neutron-diffraction experiment. Careful neutron-transmission depolarization and polarized SANS experiments have been conducted that support our theory.

## 2. Depolarization analysis for SANS   

First we need to determine the evolution of the polarization of a neutron beam traversing the sample. In general, the polarization **P**(*x*) of a spin 1/2 neutron is a vector. Assuming that the sample is homogeneous, the polarization-vector evolution can be described by a matrix 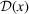, which has an exponential form, 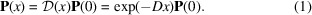
A detailed derivation and discussion of the polarization evolution is presented in the Appendix[App appa]. Similar expressions can be found in the works of Maleev & Ruban (1972 ▸) and Rosman & Rekveldt (1990 ▸)

**Figure 1:**
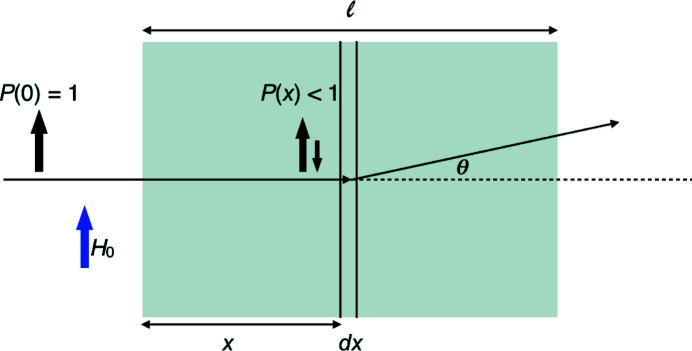
A polarized neutron beam traversing a sample of length *l* under an external magnetic field *H*_0_ is scattered at a distance *x*.

After understanding the evolution of the polarization vector, the next step is to calculate the polarized neutron cross sections. Fig. 1 ▸ illustrates that the neutron beam is scattered by the sample in a SANS experiment. Principally, the cross sections can be calculated by integrating the Blume equations (Blume, 1963 ▸) over the whole sample thickness, taking into account the evolution of the polarization vector **P**(*x*) through space. However, this is beyond the capability of a longitudinal polarization analysis. Here we make the following approximation: we choose the sample thickness so that the depolarization effect is small. The limits of this approximation will be experimentally tested and discussed (*vide infra*). With this assumption, we can still treat the neutrons as they were in defined Zeeman states of the external magnetic field, *i.e.* quasi-classical and we only consider the projection of the polarization vector on the external-field direction. Then we can approximately use a one-dimensional longitudinal polarization evolution *P*(*x*) = *P*_*z*_(*x*) = *P*(0) exp(−*Dx*), where *P* and *D* become scalars.

With this approximation we are able to derive the measured intensities of the different spin channels for a SANS experiment with longitudinal polarization analysis from the real neutron cross sections.

After a fully polarized neutron beam (assuming spin +) with an initial intensity *N*_0_ has traveled a certain distance *x* in the sample, the transmitted neutrons are depolarized to a part with spin +, *N* ^+^(*x*), and a part with spin −, *N* ^−^(*x*). Hence, we can write 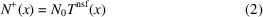
and 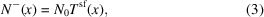
where *T* ^nsf^(*x*) and *T* ^sf^(*x*) are defined as ‘non-spin-flip’ and ‘spin-flip’ transmissions. The polarization of the transmitted beam can be expressed as 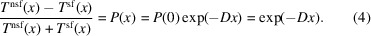
We assume that the sample under investigation has no spin-dependent absorption (otherwise it is a neutron-spin filter) and that the scattering is minor compared with the transmission. These conditions apply for most samples. Therefore we can write 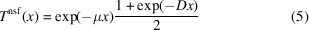
and 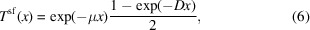
where μ is the neutron-absorption coefficient of the sample.

Based on equation (8) in the work of Brûlet *et al.* (2007 ▸), we add the contributions of the different spin states to calculate the measured spin-dependent neutron-scattering intensities *I* ^±±^ from the spin-dependent cross sections Σ^±±^. We assume that the neutron-beam optics are perfect and that there is no background. Using one of the spin channels *I* ^++^ as an example, we can write 
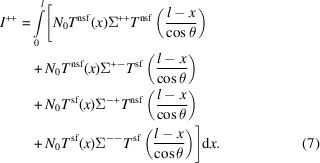
Then, we can insert equations (5) and (6) for *T* ^nsf^(*x*) and *T* ^sf^(*x*), and approximate cos θ to 1, which is a good approximation under the SANS condition (Brûlet *et al.*, 2007 ▸). The attenuation parts can be extracted from the integral 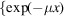 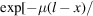 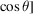 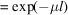 when cos θ = 1} and *I* ^++^ then becomes 
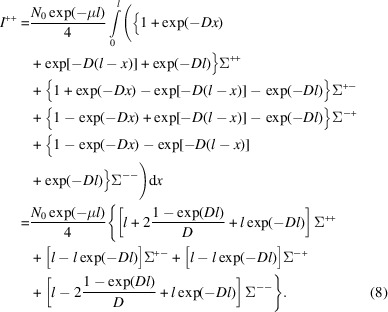
We note that exp(−*Dl*) = *P*_f_ is the final polarization after the sample that can be easily measured. Therefore *D* = −(−ln *P*_f_ /*l*), and equation (8) can be rewritten as 
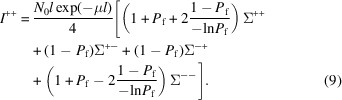
We then define the elements 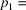 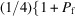 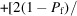 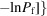, 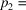 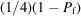 and 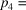 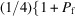 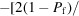 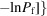. Then the intensities of all four neutron-spin channels can be written in matrix form as
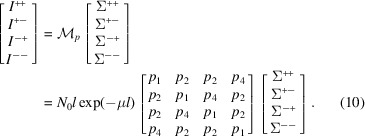
Notice that the factor *l* exp(−μ*l*) is the product of the sample length *l* and the transmission exp(−μ*l*). This factor is proportional to the scattering intensity measured in a unpolarized neutron experiment (Brûlet *et al.*, 2007 ▸) or for a sample that does not depolarize the transmitted neutron beam.

The polarized neutron cross sections are obtained from the spin-leakage corrected intensities as 
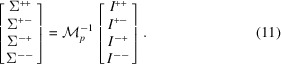
For the case of a SANS experiment without spin analysis of the scattered neutron-spin state (SANSPOL), the intensities of equation (10) reduce to 
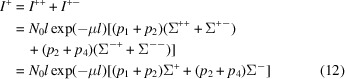
and 
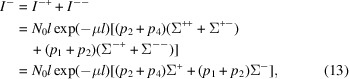
written in matrix form as
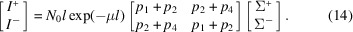
For the special case where the sample does not depolarize the transmitted beam (*e.g.* for polymer or saturated magnetic samples) *i.e.* *D* → 0, then *P*_f_ → 1 and 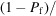 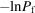 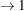. Hence, *p*_1_ → 1, *p*_2_ → 0, *p*_4_ → 0 and 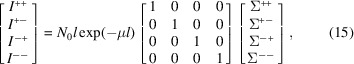
where mixing of spin channels no longer exists and thus no spin-leakage correction needs to be considered.

In polarized SANS experiments one often considers contrast measurements, *e.g.* one is interested in Σ^+^ − Σ^−^ = (Σ^++^ + Σ^+−^) − (Σ^−+^ + Σ^−−^), or Σ^+−^ − Σ^−+^. These differences between cross sections yield the intensities 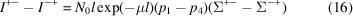
and 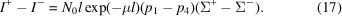
For both cases we obtain a signal strength that is proportional to 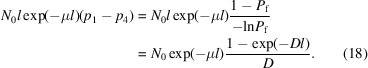
This function has a maximum when 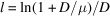 and 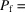 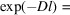 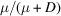. In the limit of no depolarization, *D* → 0, the signal is simply proportional to *l* exp(−μ*l*) and the optimum thickness of the sample approaches *l* → 1/μ, which corresponds to the normal 1/*e* law. This also applies to unpolarized SANS experiments.

## 3. Experimental   

To study the depolarization effect we used the commercial-grade nanocrystalline soft-ferromagnet Vitroperm as the sample (Fe_73_Si_16_B_7_Nb_3_Cu_1_), the same as investigated by polarized SANS in the work of Quan *et al.* (2020 ▸). Each sample sheet has an area of 25 × 35 mm with a thickness of 30 µm. Several stacks with different number of sheets were prepared so that the total sample thickness could be changed easily.

To check the theoretical prediction, we first performed a neutron transmission and depolarization experiment at the Super ADAM instrument (Vorobiev *et al.*, 2015 ▸) at the Institut Laue-Langevin in Grenoble, France. The incident neutron beam had a mean wavelength of λ = 5.21 Å with a wavelength spread of Δλ/λ = 0.5% and was polarized using the reflections from two supermirrors (periscope) from SwissNeutronics with an efficiency of 99.8%. The transmitted beam was analyzed by a single reflecting supermirror from SwissNeutronics with an efficiency of 99.4%. An external magnetic field **H**_0_ was applied perpendicular to the wavevector **k**_0_ using an electromagnet.

Additionally we performed a half-polarized (without analyzer) SANS measurement with the SANS I instrument (Aswal *et al.*, 2008 ▸) at the continuous spallation neutron source SINQ at the Paul Scherrer Institute, Switzerland. The incident neutron beam had a mean wavelength of λ = 5.63 Å with a wavelength spread of Δλ/λ = 10% and was polarized by a V-shaped Fe/Si supermirror transmission polarizer to *P* = 98%. In both neutron experimental setups, the neutron polarization could be reversed by means of an adiabatic spin flipper with an efficiency of 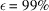. The detector was set at 11 m from the sample position with 11 m beam collimation. We used the same geometry as for the polarized SANS experiments described by Quan *et al.* (2020 ▸) and applied the external magnetic field μ_0_ **H**_0_ = 17 mT perpendicular to the wavevector **k**_0_ of the incident neutron beam and parallel to the sample’s easy axis. A 7 × 10 mm aperture defined the beam.

### 3.1. Neutron-transmission measurement   

**Figure 2:**
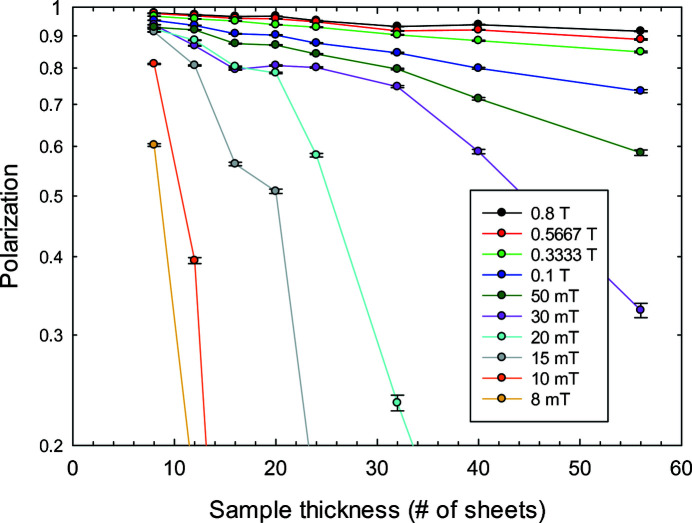
Depolarization of the transmitted neutron beam as a function of the Vitroperm sample thickness at different external magnetic fields.

We studied the depolarization as a function of sample thickness and applied magnetic field. Fig. 2 ▸ shows the final polarization on a logarithmic scale against the sample thickness for different external magnetic fields. As expected, the higher the applied field, the smaller the observed depolarization. For all field values the longitudinal polarization decays exponentially for *P* > 0.75 and then starts to decay faster. This confirms the fact that only in the case of a small depolarization can the vector polarization be approximated as a longitudinal scalar polarization, which decays exponentially. Otherwise a more complicated polarization vector needs to be taken into consideration.

### 3.2. SANS measurement   

Before the SANS measurements we performed another transmission measurement and determined the neutron-absorption coefficient of the sample, μ = 0.032 ± 0.001 sheet^−1^. Then the depolarization coefficient was measured at 17 mT with a transportable triplet dynamic nuclear-polarization spin analyzer (Quan *et al.*, 2019*b* ▸) and determined to be *D* = 0.016 sheet^−1^. We did not directly take the value determined from the transmission measurements on the Super ADAM instrument since the incident neutron wavelengths for the two measurements were different. However, the values are in good agreement.

**Figure 3:**
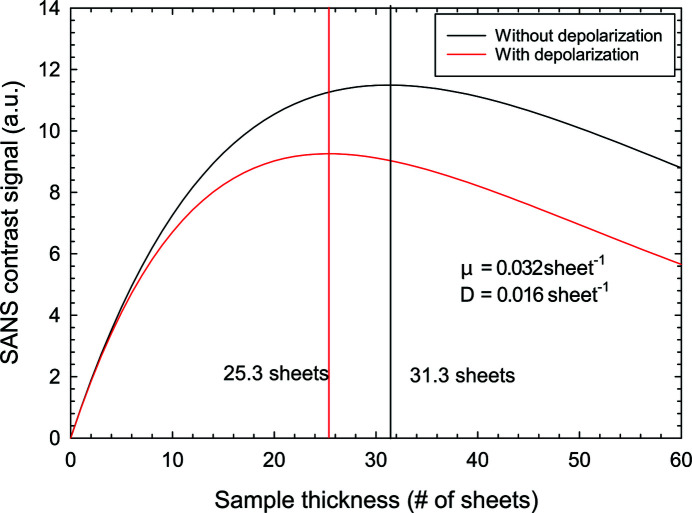
Simulation of the Vitroperm SANS contrast signal as a function of the sample thickness, with and without depolarization. For the comparison, the parameters determined by the transmission depolarization measurement at 17 mT are taken: μ = 0.032 sheet^−1^ and *D* = 0.016 sheet^−1^. Considering the depolarization, the optimum thickness shifts from 1/μ = 31.3 to 25.3 sheets.

The experimental values of the absorption and depolarization coefficients can be used to evaluate equation (18), as shown in Fig. 3 ▸, which can be compared with the case without depolarization, *D* = 0. Taking the depolarization into account, the optimum sample thickness to obtain the highest contrast signal is *l* = 25.3 sheets, *i.e.* less than the 1/μ = 31.3 sheets for the case of no depolarization. However, ∼18 sheets of the sample will depolarize the neutron beam to *P* = 75%. For larger thicknesses the neutrons should get depolarized even faster. We therefore expect that the signal maxima should be reached for less than 25 sheets and decrease faster than the model predicts.

**Figure 4:**
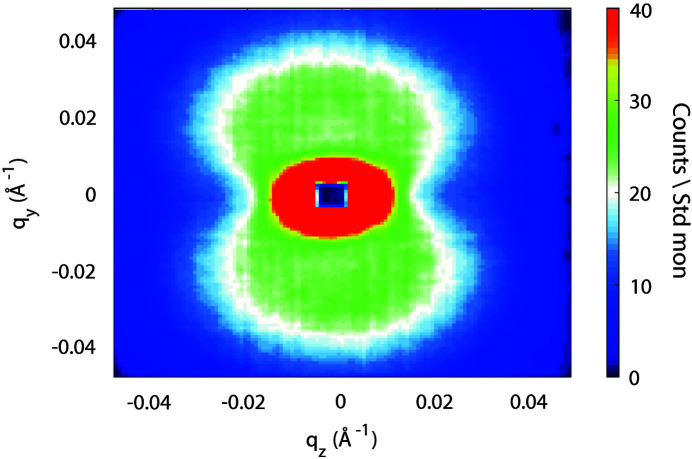
An example of the sum of the two spin-polarized SANS intensities *I* ^+^ + *I* ^−^ with 35 sheets of Vitroperm. This is equivalent to an unpolarized SANS intensity.

**Figure 5:**
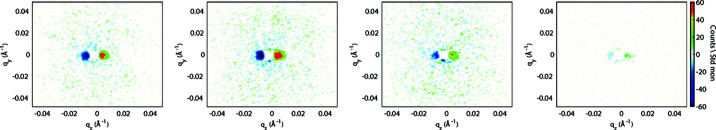
Sample-thickness dependence of the polarized SANS contrast signal *I* ^+^ − *I* ^−^. Sample thickness from left to right: 7, 19, 26 and 35 sheets of Vitroperm (Fe_73_Si_16_B_7_Nb_3_Cu_1_).

We performed half-polarized SANS measurements at 17 mT as a function of the sample thickness. The sample was always first saturated at 0.8 T and then brought to 17 mT in order to follow the same hysteresis curve. The scattering patterns observed were similar to those presented in Fig. 4 in the work of Quan *et al.* (2020 ▸). Fig. 4 ▸ shows an example of the sum of the two spin-polarized SANS intensities *I* ^+^ + *I* ^−^ (equivalent to the unpolarized SANS intensity) with 35 sheets of Vitroperm, while Fig. 5 ▸ shows the difference between the two spin-polarized SANS intensities *I* ^+^ − *I* ^−^ with 7, 19, 26 and 35 sheets of Vitroperm. In Fig. 5 ▸, the strong left–right asymmetric signal at low scattering vector *q* originates from the chiral interaction caused by the defect-induced Dzyaloshinskii–Moriya interaction (DMI) (Michels *et al.*, 2019 ▸; Quan *et al.*, 2020 ▸), while the up–down weak signal arises from the nuclear-magnetic interference scattering. We observe that with 19 sheets of sample both the asymmetric DMI and the nuclear-magnetic interference signals are much stronger than with 26 and 35 sheets where stronger unpolarized signals [*I* ^+^ + *I* ^−^ ∝ *l* exp(−μ*l*)] should be expected.

**Figure 6:**
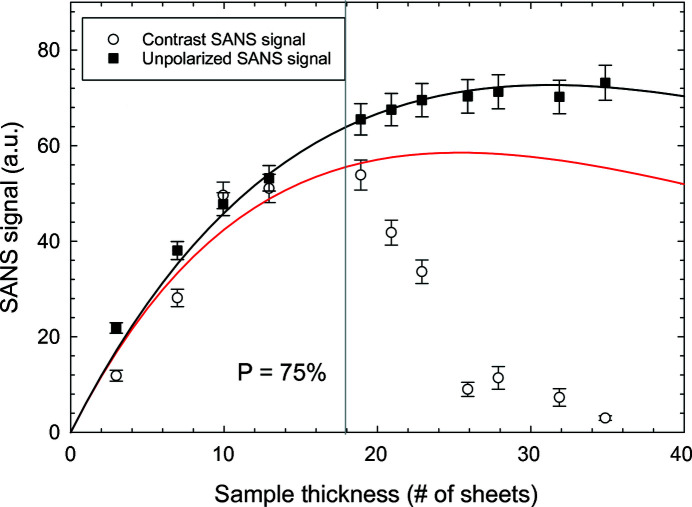
Normalized contrast *I* ^+^ − *I* ^−^ and the unpolarized *I* ^+^ + *I* ^−^ scattering intensities as a function of the sample thickness. The unpolarized intensity *I* ^+^ + *I* ^−^ is fitted to *l* exp(−μ*l*) (with fixed μ = 0.032 sheet^−1^ and only one free parameter: the scaling factor), drawn as the black solid line. The first five data points of the contrast SANS signal (the polarization for the fifth data point is calculated to be *P* = 74%) are fitted to equation (18) (with fixed μ = 0.032 sheet^−1^, *D* = 0.016 sheet^−1^ and only one free parameter: the scaling factor), drawn as the red solid line.

The normalized contrast *I* ^+^ − *I* ^−^ and the unpolarized *I* ^+^ + *I* ^−^ scattering intensities as functions of sample thickness are shown in Fig. 6 ▸. For the contrast intensity *I* ^+^ − *I* ^−^ we have plotted the sum of the positive signal at positive *q*_*z*_ and the modulus of the negative signal at negative *q*_*z*_ in the two horizontal sectors (0.006–0.015 Å^−1^, ± 25°, see Fig. 5 ▸). For the unpolarized intensity *I* ^+^ + *I* ^−^, we summed up the neutron counts in the *q* range of 0.023–0.045 Å^−1^. The error bars of all the data points consider both the statistical error and an estimated 5% systematic error. We expect that the systematic error originates from the differences between different sheets of the sample and their alignment. The unpolarized intensity *I* ^+^ + *I* ^−^ follows the function *l* exp(−μ*l*) (same as *D* = 0), plotted as the black solid line where μ = 0.032 sheet^−1^ and the scaling factor was fitted. For the contrast measurements, we fitted the first five data points to equation (18) with fixed μ = 0.032 sheet^−1^ , *D* = 0.016 sheet^−1^ and a free scaling factor, which is drawn as the solid red line. The polarization for the fifth data point is calculated to be exp(−*Dl*) = 0.74. The model can describe the scattering intensities well for a sample of a thickness that does not significantly depolarize the transmitted neutron beam, *P* > 0.75 (the longitudinal polarization still decays as an exponential function). As discussed, for thicker samples the polarization decays faster than exponential, and as a consequence we expect that the optimum contrast signal should be reached with less than the calculated 25.3 sheets of sample and decrease faster than the model predicts. However, we notice that this effect is much stronger than expected and the signal already peaks at ∼19 sheets and then starts to decrease extremely fast. This fast decrease is also confirmed by evaluating the up–down nuclear-magnetic interference signal and is directly visible in Fig. 5 ▸. This strongly emphasizes the importance of considering the depolarization of the neutron beam traversing the sample and optimizing the sample thickness in a neutron-scattering experiment, which may be decisive for being able to observe a signal.

## 4. Results and outlook   

We have addressed a fundamental problem to be considered when performing polarized neutron experiments: the transmitted neutron beam is depolarized by the sample, in particular by ferromagnetic samples, and as a consequence the measured polarized cross sections are actually contaminated by the other spin channels. To address this problem, we have developed a model describing the evolution of the polarization through the sample. Based on this model we are able to calculate the scattered neutron intensities of polarized SANS experiments in the limit of small depolarization (*P* > 0.75). This allows us to correct the contamination from the other spin channels and optimize the sample thickness for the neutron experiments. The model has been verified experimentally by a neutron-transmission measurement and a polarized SANS measurement. We showed that it is essential to consider the depolarization effect and optimize the sample thickness accordingly. Furthermore, the depolarization effect and our approach are not limited to SANS. The model can be tailored according to the experimental geometry and the sample shape in other types of neutron-diffraction experiments. We suggest that the depolarization of a sample, in particular if it is a ferromagnet, should be characterized before any polarized neutron experiment is performed. Ideally, every neutron instrument for polarized neutron experiments should be equipped with a spin analyzer to monitor the depolarization by the sample in case of changing experimental conditions.

The model falls short in describing the scattering of thick samples that significantly depolarize the transmitted neutron beam. Under these conditions, we observed a much more prominent depolarization effect. Then the vector form of the polarization as well as the full Blume equation (Blume, 1963 ▸) need to be considered, which is certainly beyond the capability of a typical neutron instrument with longitudinal polarization analysis. 

## Appendix A Polarization evolution during neutron transmission

In general, the polarization **P**(*x*) of a spin 1/2 neutron is a vector and we may write its evolution as

where  is a 3 × 3 matrix denoting the depolarization. We assume that the sample is homogeneous. Then the polarization evolution only depends on the neutron path length in the sample,

It is natural that the depolarization should satisfy the following relations:

and

where *I* is the identity matrix.

Since the evolution of the polarization vector should be a continuous process,  should be continuous. Using these three relations, we can write

thus

where  is a constant matrix. We can easily solve this differential equation and show that the evolution matrix can be written as an exponential function:

Here we insert a ‘−’ sign to denote the depolarization. Thus we obtain the evolution of the polarization vector:

which is equation (1). We can make the following remarks. Mathematically, when  satisfies the equations (21), (22) and (23), it belongs to the continuous translation group in one dimension (Tung, 1985 ▸), and equation (26) follows directly. Physically, this means that not just the length but also the direction of the polarization vector **P**(*x*) evolves in space. In other words, the neutrons can go from the initially defined Zeeman states to mixed states. Similar expressions for the depolarization matrix (and the polarization vector) have been derived in the works of Maleev & Ruban (1972) ▸ and Rosman & Rekveldt (1990 ▸).

We realize that equation (1) is very similar to the solution of the Bloch equations for a time-independent Hamiltonian (Bloch, 1946 ▸), which describe the evolution of the polarization vector in magnetic resonance. In analogy, here the space *x* is directly connected to time *t* via *x* = *vt*, where *v* is the fixed neutron speed. Assuming *D* can be diagonalized, equation (22) implies that  can be diagonalized for all *x* by the same matrix *T*, . Here  is a diagonal matrix and *T* is independent of *x*. The diagonalization is equivalent to a transform to the principal axes. From equations (21) and (23) it follows that  is a matrix where all three diagonal elements are exponential functions, , where *D*_d_ is an *x*-independent diagonal matrix. Then the evolution matrix becomes     , where *D* = *T* ^−1^ *D*_d_ *T* is an *x*-independent matrix. The real part of *D*_d_ corresponds to relaxation while the imaginary part corresponds to precession (phase shift).
